# Our Medical Literature

**Published:** 1881-12

**Authors:** John S. Billings

**Affiliations:** Surgeon U. S. Army


*International Medical Congress*, 1881.

### OUR MEDICAL LITERATURE.

ADDRESS BY JOHN S. BILLINGS, M. D., SURGEON U. S. ARMY.

When I say "Our Medical Literature," it is not with reference to that of any particular country or nation, but to that which is the common property of the educated physicians of the world as represented here to-day—the literature which forms the intra- and inter-national bond of the medical profession of all civilized countries; and by virtue of which we, who have come hither from the far West and the farther East, do not now meet, for the first time, as strangers, but as friends, having common interests, and though of many nations, a common language, and whose thoughts are perhaps better known to each other than to some of our nearest neighbors.

It is usual to estimate that about one-thirtieth part of the whole mass of the world's literature belongs to medicine and its allied sciences. This corresponds very well to the results obtained from an examination of bibliographies and catalogues of the principal medical libraries. It appears from this that our medical literature now forms a little over 120,000 volumes properly so called, and about twice that number of pamphlets, and that this accumulation is now increasing at the rate of 1500 volumes and 2500 pamphlets yearly.

Let us consider the character of this annual growth, somewhat in detail, first giving some figures as to the number of those who are producing it.

There are at the present time scattered over the earth about 180,000 medical men, who, by a liberal construction of the phrase, may be said to be educated; that is, who have some kind of a diploma, and for whose edification this current medical literature is produced. Of this number about 11,600 are producers of, or contributors to, this literature, being divided as follows:—United States, 2,800; France and her colonies, 2,600; the German Empire and Austro-Hungary, 2,300; Great Britain and her colonies, 2,000; Italy, 600; Spain, 300; all others, 1,000. These figures should be considered in connection with the number of physicians in each country; but this I can only give approximately, as follows:—United States, 65,000; Great Britain and her colonies, 35,000; Germany and Austro-Hungary, 32,000; France and her colonies, 26,000; Italy, 10,000; Spain, 5,000; all others, 17,000.

It will be seen from these figures that the number of physicians who are writers is proportionately greatest in France, and least in the United States. As regards France, this is largely due to the requirement of a printed thesis for graduation, which of itself adds between six and seven hundred annually to the number of writers.

Excluding popular medicine, pathies, pharmacy, and dentistry, all of which were included in the figures for the annual product just given, we find that the contributions to medicine, properly so called, form a little over 1,000 volumes and 1,600 pamphlets yearly.

For 1879, "Rupprecht's Bibliotheca" gives as the total number of new medical books, excluding pamphlets, periodicals, and transactions, 419; divided as follows, viz: France, 187; Germany, 110; England, 43; Italy, 32; United States, 21; all others, 26. These figures are, however, too small, and especially so as regards Great Britain and the United States. The "Index Medicus" for the same year shows by analysis that the total number of medical books and pamphlets, excluding periodicals and transactions, was 1643; divided as follows: France, 541; Germany, 364; United States, 310; Great Britain, 182; all others, 246. This does not include the inaugural thesis, of which 693 were published in France alone.

The special characteristics of the literature of the present day are largely due to journals and transactions, and this is particularly true in medicine. Our periodicals contain the most recent observations, the most original matter, and are the truest representations of the living thought of the day and of the tastes and wants of the great mass of the medical profession, a large part of whom, in fact, read very little else. They form about one-half of the current medical literature, and in the year 1879 amounted to 655 volumes, of which the United States produced 156; Germany, 129; France, 122; Great Britain, 54; Italy, 65; and Spain, 24. This is exclusive of journals of pharmacy, dentistry, etc., and of journals devoted to medical sects and isms. These are given in an appended table, from which it appears that the total number of volumes of medical journals and transactions of all kinds was for the year 1879, 850; and for 1880, 864. The figures for 1880 are too small, but the real increase is slight. During the year 1879, the total number of original articles in medical journals and transactions which were thought worth noting for the "Index Medicus" was a little over 20,000. Of these there appeared in American periodicals 4781; in French, 4608; in German, 4,027; in English, 3,592; in Italian, 1,210; in Spanish, 703; in all others, 1,248. The figures for 1880 are about the same. It will be seen that at present more of this class of literature appears in the English language than in any other, and that the number of journal contributions is greatest in the United States. The actual bulk of periodical literature is, however, greatest in Germany, owing to the greater average length of the articles. With regard to the mode of publication, I will only say that in all countries except Spain the greater number of medical periodicals are monthly, while in Spain they are semi-monthly. It is this periodical literature which, more than anything else, makes medicine cosmopolitan; and although, as regards new discoveries or methods of treatment, it is still somewhat farther from London or Berlin or Paris to New York than it is from New York to either of these places, the discrepancy is gradually becoming less.

Many of the medical journals are very short-lived, but the total number is increasing. In 1879, twenty-three such journals ceased, but sixty new ones appeared, and in 1880 there were twenty-four deaths and seventy-eight births in this department of literature. Over one-third of this fluctuation occurs in the United States alone, France being next in the scale, Spain third, and Italy fourth, while Great Britain is the most stable of all.

This merely quantitative classification gives, of course, no idea as to the character, and very little as to the value, of the product. Let us now consider it by subjects. During 1879 there were published 167 books and pamphlets, and 1543 articles relating to anatomy, physiology, and pathology—that is, to the biological or scientific side of medicine. Dividing this again by nations, we find that Germany produced a majority of the whole. France being second. The proportionate production by nations of this class of literature is perhaps better shown by an analysis of the bibliography of physiological literature for the year 1879, as published by the *Journal of Physiology*. This shows 59 treatises and 500 articles in German, 17 treatises and 227 articles in French, 5 treatises and 77 articles from Great Britain, 8 treatises and 41 articles from Italy, and 2 treatises and 24 articles from the United States. The number of authors for this product was—German, 393; French, 119; English, 59; Italian, 39; United States, 19; all others, 41. For the year 1880 the same journal reports 62 treatises and 452 articles from Germany, 23 treatises and 216 articles from France, 12 treatises and 76 articles from Great Britain, 4 treatises and 51 articles from Italy, 6 treatises and 25 articles from the United States, and 10 treatises and 31 articles from all other countries.(a)

(a)NOTE.—The difference between these figures and those of the "Index Medicus" is due, on the one hand, to the fact that the *Journal of Physiology* includes articles which are placed under other headings in the

When we turn to the literature of the art, or practical side of the profession, the figures are decidedly different. We find over 1,200 treatises and 18,000 journal articles which come under this head, and the order of precedence of countries as to quantity is--France, United States, Germany, Great Britain, Italy, and Spain. The appended tables give still further subdivisions, showing by nations the number of works and journal articles upon the practice of medicine, surgery, obstetrics, hygiene, etc., for the years 1879 and 1880, and some of the figures will be found interesting. A marked increase has occurred in the literature of hygiene during the last two years, and this especially in England, France, Germany and the United States. The literature of diseases of the nervous system, of opthalmology, otology, dermatology, and gynæcology, is also increasing more rapidly than that of the more general branches.

It would of course be extremely unscientific to use these figures as if they represented positively ascertained and comparable facts, the accuracy of which, as well as of the classification, could be verified. They represent merely the opinions of an individual--first as to whether each treatise or pamphlet included in these statistics was worth noting, and second as to how it should be classed. Had everything been indexed, the figures, for journal articles at least, might have been nearly doubled; while if the selection had been made by a more severe critic they might have been reduced one-half.

If I had to do the work again I should not obtain the same results. The prevailing error is that, as regards journal articles, the figures are too large, for some of those included are of so little value or interest that they are, I fear, never read by more than two persons.

Be that as it may, I think we can take them as indicating certain differences in the direction of work of the medical authors of the great civilized nations of the earth; but they must be considered as approximations only; and the statistical axiom

"Index Medicus," and on the other hand to the fact that the *Journal* has a different standard of excellence from that of the "Index," rejecting many articles which the latter must accept as original.

must be remembered that the results obtained from a large number of facts are applicable to an aggregate of similar facts, but not to single cases. There will be a certain number of medical books and papers printed next year, just as there will be a certain number of children born; and as we can within certain limits predict the number of these births and the proportion of the sexes, or even of monsters,—so we can within certain limits predict the amount and character of the literature that is yet to come, the ideas that are yet unborn. The differences are due to race, politcal organization, and density of population. As Dr. Chadwick has pointed out, in speaking of the statistics of obstetric literature, one of the chief causes of the multiplication of medical societies is geographical. "In England it is possible for those who are specially interested in gynæcology and obstetrics to attend the meetings of the Obstetrical Society of London, whereas in America the distances are so great that this is impossible." Speaking broadly we may say that at present Germany leads in scientific medicine, both in quantity and quality of product, and that the rising generation of physicians are learning German physiology. But the seed has gone abroad, and scientific work is receiving more and more appreciation everywhere.

Seven years ago Professor Huxley declared that if a student in his own branch showed power and originality he dared not advise him to adopt a scientific career, for he could not give him the assurance that any amount of proficiency in the biological sciences would be convertible into the most modest bread and cheese. To-day I think he might be bolder, for such a fear would hardly be justifiable; at all events, in America, where such a man as is referred to could almost certainly find a place, bearing in mind the Professor's remark that it is no impediment to an original investigator to have to devote a moderate portion of his time to giving instruction either in the laboratory or in the lecture-room.

Within the last ten years the literature of France, Germany, Great Britain, and the United States has contained much with regard to medical education and the means for its improvement. In all these countries there is more or less dissatisfaction with the existing condition of things, although there is no general agreement as to the remedy. Solomon's question, "Wherefore is there a price in the hands of a fool to get wisdom, seeing he hath no heart to it?" is now easily answered, for even a fool knows that he must have the semblance of wisdom, and a diploma to imply it, if he is to succeed in the practice of medicine; but to insure the value of a diploma as a proof of education is the difficulty.

This evidence of discontent and tendency to change is a good sign. In these matters stillness means sleep or death—and the fact that the stream is continually changing its bed shows that its course lies through fertile alluvium, and not through sterile lava or granite.

I have said that as regards scientific medicine we are at present going to school to Germany. This, however, is not the case with regard to therapeutics, either external or internal—in regard to which I presume that the physicians of each nation are satisfied as to their own pre-eminence. At all events, it is true that, for the treatment of the common diseases, a physician can obtain his most valuable instruction in his own country; among those whom he is to treat. Just as each individual is in some respects peculiar and unique, so that even the arrangement of the minute ridges and furrows at the end of his forefinger differs from that of all other forefingers, and is sufficient to identify him; and as the members of certain families require special care to guard against hemorrhage, or insanity, or phthisis; so it is with nations and races. The experienced military surgeon knows this well, and in the United States, which is now the great mixing ground, illustrations of race-peculiarities are familiar to every practitioner.

Neither the tendency nor the true value of this current medical literature can be properly estimated by attending to it alone. It is a part of the thought of the age—of that wonderful kaleidoscopic pattern which is unrolling before us—and must be judged in connection with it. From several sources of high authority there have come of late years warnings and laments that science is becoming too utilitarian. For example, Professor

Du Bois-Reymond, in his address upon civilization and science, says that that side of science which is connected with the useful arts is steadily becoming more prominent, each generation being more and more bent on material interests. "Amid the unrest which possesses the civilized world, men's minds live, as it were, from hand to mouth. . . . . And if industry receives its impulse from science, it also has a tendency to destroy science. In short, idealism is succumbing in the struggle with realism, and the kingdom of material interests is coming." Having laid down this rather pessimistic platform, he goes on to state that this is especially the case in America, which is the principal home of utilitarianism, and that it has become the custom to characterize as "Americanization" the dreaded permeation of European civilization by realism. If this characterization be correct, it would seem that Europe is pretty thoroughly Americanized as regards attention to material interests and appreciation of practical results. But the truth of the picture seems to me doubtful. Science is becoming popular, even fashionable; and some of its would-be votaries rival the devotees of modern Æstheticism in their dislike and fear of the sunlight of comprehensibility and common sense. The languid scientific swell, who thinks it bad style to be practical, who takes no interest in anything but pure science, and makes it a point to refrain from any investigations which might lead to useful results, lest he might be confounded with mere "practical men" or "inventors," exists, and has his admirers. We have such in medicine, and their number will increase.

The separation of biological study from practical medicine, which has of late years become quite marked in the literature of the subject, has its advantages and disadvantages. Thus far the former have far outweighed the latter, and both the science and the art of medicine have been promoted thereby. But are not the physiologists, or as I believe they prefer to be called, the biologists, separating themselves too completely from medicine for the best interests of their own science, in that they are neglecting human pathology? In our hospital wards and among our patients, nature is continually performing experiments which the most dexterous operator cannot copy in the laboratory—she is, as Professor Foster says, "a relentless and untrammelled vivisector, and there is no secret of the living frame which she has not, or will not, at some time or place, lay bare in misery and pain."

TO BE CONTINUED.

